# Does the time spent in retirement improve health? An IV-Poisson assessment on the incidence of cardiovascular diseases

**DOI:** 10.1016/j.socscimed.2024.117084

**Published:** 2024-08

**Authors:** Dario Fontana, Chiara Ardito, Roberto Leombruni, Elena Strippoli, Angelo d’Errico

**Affiliations:** a Epidemiology Unit ASL TO3, Grugliasco, (TO), Italy; b European Commission, Joint Research Centre (JRC), Ispra, Italy; c Department of Economics and Statistics “Cognetti de Martiis”, University of Torino, Torino, Italy

## Abstract

In recent decades, pension reforms have been implemented to address the financial sustainability of social security systems, resulting in an increase in the retirement age. This adjustment has led to ongoing debates about the relationship between retirement and health.

This study investigates the impact of time spent in retirement on the risk of cardiovascular disease (CVD) in Italy. It uses a comprehensive dataset that includes socioeconomic, health, and behavioural risk factors, which is linked to administrative hospitalisation and mortality registers. To address the potential endogeneity of retirement, we employ an instrumental variables approach embedded in a Poisson rate model.

The results show that, on average, years spent in retirement have a beneficial effect on the risk of CVD for both men and women. Each additional year spent in retirement reduces the incidence of such diseases by about 17% for men and 29% for women. Stratified analyses and robustness tests show that the benefits of retirement appear to be more robust and pronounced in men and in certain groups, particularly men in manual occupations or with poor ergonomic conditions at work.

These results highlight that delaying access to retirement may lead to an increased burden of CVD in the older population. In addition, the protective effect of retirement on the development of CVD among workers with poorer ergonomic conditions underlines the different impact of increasing the retirement age on different categories of workers and the need for targeted and differentiated policies to avoid hitting the more vulnerable.

## Highlights

• This study uses advanced IV-Poisson methods to assess the impact of years of retirement on health in Italy.
• More years of retirement reduce the incidence of CVD, suggesting a protective effect in both sexes.
• Men with lower occupational class and poor ergonomic conditions show the most pronounced and robust effects.
• This article contributes to the discussion on future pension reforms and retirement age differentiation.
• More attention needs to be paid to the health and socioeconomic conditions of older workers.

## 1 Introduction

Life expectancy has increased due to advances in medicine and better living standards. However, the combination of longer lives, lower birth rates, and early retirement trends in the 1980s and 1990s has put financial pressure on welfare systems (Gruber and Wise, 2007). This led to pension system reforms in European countries in the 1990s, aiming to limit early retirement and raise the age for pension eligibility. Most European countries have set the normal pension age at 67, and further increases are expected, especially in countries with automatic adjustment mechanisms linking pension age to changes in life expectancy. This means retirement age in these countries will likely exceed 67 soon (OECD, 2019).

In this time of significant changes, there is concern that raising the retirement age may prolong exposure to workplace psychological and physical strain, which could negatively impact health or force vulnerable workers into other welfare programs such as long-term sick leaves, disability pensions, or unemployment (Hanel and Riphahn, 2012; Ardito, 2021; Soosaar et al., 2021).

The existing evidence on the relationship between pension reforms, retirement, and health has yielded mixed results, as demonstrated by recent meta-analyses focusing on the impact of retirement on depressive disorders (Odone et al., 2021), cardiovascular health (Xue et al., 2020), and on a variety of health outcomes (Filomena and Picchio, 2023; Garrouste and Perdrix, 2022). This divergence may be due to variations in research design (Nishimura et al., 2018) and in the definition of exposure and outcome variables (Filomena and Picchio, 2023; Garrouste and Perdrix, 2022).

Prior studies have been hindered by methodological issues related to the endogeneity of retirement with respect to health, including potential reverse causality, which may have led to an underestimation of the true link between retirement and post-retirement health (Han, 2021). While recent studies have attempted to address endogeneity bias, their findings also remain inconclusive as shown by some literature reviews in this area (Nishimura et al., 2018; Filomena and Picchio, 2023; Garrouste and Perdrix, 2022). In the latest meta-analysis by Filomena and Picchio (2023), the majority of the included studies (60%) found no statistically significant effect, while 28% reported that retirement improved health, and the remaining 12% found that retirement was detrimental to health. However, these studies assessed a wide array of health outcomes (such as mental health, physical health, health care utilization, mortality, cognitive abilities, etc.), which could be problematic as retirement may affect different health dimensions in different ways (Hasselhorn et al., 2022; Nishimura et al., 2018; Garrouste and Perdrix, 2022). For instance, the positive impact of retirement on self-reported health and mental health is a well-established result supported by various literature reviews (Nishimura et al., 2018; Garrouste and Perdrix, 2022; van der Heide et al., 2013), as well as a meta-analysis specifically focused on depression (Odone et al., 2021). Conversely, as pointed out by Garrouste and Perdrix (20–22), retirement is generally associated with a negative impact on cognitive ability, with evidence suggesting that cognitive decline may accelerate after retirement, especially for workers exposed to better working conditions and stimuli, such as white collar and higher skilled workers. (Mazzonna and Peracchi, 2017). What the effect of retirement on mortality and morbidity is appears still equivocal, even restricting the attention only to studies that adopt an explicit causal design (Garrouste and Perdrix, 2022), as both null (Hernaes et al., 2013; Hagen, 2018; Bozio et al., 2021), beneficial (Ardito et al., 2020; Hallberg et al., 2015), and detrimental effects (Kuhn et al., 2020; Behncke, 2012) have been reported. Another factor contributing to the inconsistency may be the nature of retirement (Hasselhorn et al., 2022), as involuntary or mandatory retirement has been found to have a more negative impact on subsequent health compared to voluntary retirement (Dave et al., 2008; Van Solinge and Henkens, 2007; Filomena and Picchio, 2023). Another source of variation in the results outlined in the literature may be attributed to differences in national retirement legislation. This can include variations in statutory pension age, access to early retirement, and other factors that potentially influence workers' decisions and possibility to retire, and access to other welfare programs like disability pension, unemployment, and long-term sick leave (Hasselhorn and Apt, 2015). Finally, some studies find evidence of heterogeneous effects by gender, health, and type of occupation. For example, the findings of Eibich (2015), Ardito et al. (2020) and Carrino et al. (2023) suggest that workers exposed to highly physically demanding jobs may benefit from retirement, while workers in highly skilled occupations remain unaffected or even worse off (Mazzonna and Peracchi, 2017). However, there are still relatively few studies on this aspect and the issue remains a priority for future research (as suggested by Filomena and Picchio, 2023; Garrouste and Perdrix, 2022).

This study examines the impact of retirement on cardiovascular health, leveraging the variation in retirement timing between cohorts resulting from pension reforms that raised normal and early pension age. The analysis focuses on Italy and utilizes a comprehensive dataset containing information on socioeconomic status, health, and behavioural risk factors, linked to official administrative hospitalisation and mortality records spanning multiple years. To address the endogeneity of retirement, an instrumental variable approach embedded in a Poisson GMM model is employed. The study considers as the primary outcome cardiovascular disease (CVD), a group of disorders that includes coronary heart disease, cerebrovascular disease and other diseases of the circulatory system, which is a leading cause of death and hospitalisation in Italy and worldwide (WHO - World Health Organization, 2020), and a major contributor to global health loss for both sexes (Vos et al., 2020). The CVD group of diseases is particularly relevant in the context of retirement and reforms aimed at delaying retirement, as physically and psychologically demanding working conditions, acting through behavioural and pathophysiological processes, have been widely associated with the development and increased risk of CVD (Marmot et al., 1997; Perk et al., 2012; Holtermann et al., 2021).

Our article makes several contributions to the existing literature. Firstly, we delve into the underexplored area of retirement's effects on CVD, where evidence from retirement and pension reform's impact on extending working life remains inconclusive (Xue et al., 2020). Secondly, our study contributes methodologically by employing an advanced statistical method (IV-Poisson GMM) to address the endogeneity of retirement and model the time elapsed since retirement to the occurrence of CVD. This approach combines the strengths of methods conventionally used in different disciplines, providing a rigorous way to tackle endogeneity and model disease occurrence over time. Thirdly, our study benefits from high-quality and representative data, drawing on CVD outcomes from administrative hospitalisation and mortality registers linked to a national sample survey. This enables accurate information on outcomes, generalizability to the entire Italian population of corresponding age, and control for individual work, health, and lifestyle characteristics. Lastly, our study goes beyond previous research by examining the potential effects of retirement on health beyond the mean. We assess effect modifications by occupational social class and ergonomic conditions at baseline, particularly focusing on the physical strain of the job prior to retirement. This analysis incorporates exposure to ergonomic factors through a Job-Exposure Matrix specifically calibrated for the Italian workforce (Fontana et al., 2022), providing a detailed description of the physical workload experienced by retirees before retirement.

The study's findings indicate that, on average, retirement has a protective effect on CVD risk for both men and women, with an additional year in retirement leading to a significant reduction in CVD incidence by approximately 17% in men and 29% in women. Moreover, the analysis indicates that the impact of the time spent in retirement on CVD appears to be more robust for men and more pronounced among retirees previously employed in manual occupations with poorer ergonomic conditions. This suggests that the primary mechanism through which retirement influences cardiovascular health is the relief from manual work and, specifically, exposure to ergonomic factors.

The remaining sections of the paper are structured as follows: Sections 2, 3 provide details on the data and empirical strategy, section 4 presents the results and several sensitivity checks, section 5 discusses the findings in relation to previous literature, and section 6 concludes the paper.

## 2 Data and materials

### 2.1 Data

For this analysis, we have used a dataset derived from the linkage of administrative health data with survey data, the Italian Longitudinal Study (ILS).^1^ The ILS is derived from the 2005 National Health Interview Survey (NHIS), which was conducted by the National Institute of Statistics (ISTAT) and aimed to collect detailed information on: health conditions, chronic diseases, disabilities, lifestyle, and the use of health services, as well as information on individual and family socio-economic characteristics. The 2005 NHIS was conducted on a representative sample of the Italian resident population of 50,474 households and 128,040 individuals. Subjects who participated in NHIS 2005 were followed up for mortality and morbidity, from the year of the survey, until December 31, 2014 b y means of deterministic record-linkage with the National Mortality Register of the Italian National Institute of Statistics and the National Database of Hospital Discharges of the Italian Ministry of Health.

### 2.2 Sample selection

For our analysis, we select all individuals who reported in the 2005 NHIS survey that they were retired and had worked as salaried employees in the private sector in their last job. First, only retirees who answered questions about their last job were included. We then restricted the sample to individuals who reported a retirement age at least equal to the age of first eligibility for statutory early retirement, based on the relevant birth cohort, gender, and sector of activity (private), ranging from 50 to 57 over the period of analysis (Supplementary Data Table 1, Table 2). Very old individuals were excluded, using as upper age limit the gender-specific average life expectancy in 2005 (i.e., 78 for men and 83 for women). The applied selection criteria were designed to identify individuals transitioning from their last job into retirement, excluding those who are long-term unemployed, discouraged workers, or otherwise inactive. Although there is still a possibility that some of the retirees in the sample could have ceased working for unobservable reasons not directly related to career end, our analysis based on an instrumental variable strategy addresses these potential sources of endogeneity biases.

**Table 1:** Descriptive statistics of the analytical sample.

	**Men**	**Women**
**Number of subjects** (N)	4061	1958
**Days of follow - up** (mean, sd)	2787 (1111)	2979 (995)
**Cumulative CVD incidence** (%)	29.6	23.3
$DistR_{it}$ Years in retirement (mean, sd)	10.0 (6.0)	13.4 (7.4)
**Age at 2005** (mean, sd)	68.2 (5.1)	70.4 (6.3)
**BMI (%)**		
Normal weight or underweight	34.3	50.4
Overweight	51.6	35.3
Obese	14.1	14.3
**PCS** (mean, sd)	48.5 (9.1)	44.9 (10.3)
**Pack-years of smoking** (%)		
0 (never smoker)	34.9	80.4
0.1–20	18.7	10.7
>20	46.4	8.8
**Leisure-time physical activity** (%)		
No activity	44.1	58.5
Light	37.1	32.6
Regular or intense	18.8	8.9
**Geographical area** (%)		
North-West	27.1	28.3
North-East	21.2	24.7
Centre	20.2	20.5
South and Islands	31.5	26.5
**Socio-occupational class** (%)		
Bourgeoisie or middle class	24.9	18.2
Manual workers	75.1	81.8
**Ergonomic index** (median, 25°–75°)	36.1 (24.7–43.7)	34.5 (29.0–43.7)
Subjects with missing ergonomic index	851	296

**Table 2:** Incidence rate ratios (IRR) and 95% Confidence Interval of cardiovascular diseases (CVD) associated with years in retirement: IV Poisson GMM, by gender.

	Men (N 4061)			Women (N 1958)		
	**Model 1**	**Model 2**	**Model 3**	**Model 1**	**Model 2**	**Model 3**
	**IRR**	**IRR**	**IRR**	**IRR**	**IRR**	**IRR**
$DistR_{it}$ Years in retirement	0.80**	0.82**	0.83**	0.65**	0.67**	0.71**
(95% CI)	(0.72–0.89)	(0.72–0.93)	(0.74–0.93)	(0.55–0.77)	(0.57–0.79)	(0.60–0.84)
**Age**	1.26**	1.23**	1.22**	1.41**	1.42**	1.39**
**BMI**						
Normal weight or underweight (ref.)		1	1		1	1
Overweight		0.98	0.97		1.31	1.01
Obese		1.17	1.15		0.89	0.92
**PCS**		0.98**	0.98*		1.01	1.00
**Pack-years of smoking**						
0 never smokers (ref.)		1	1		1	1
0.1–20		1.09	1.07		1.02	0.96
>20		1.21**	1.22**		0.97	1.03
**Leisure-time physical activity**						
No activity (ref.)		1	1		1	1
Light		1.19*	1.16*		1.28	1.41*
Regular or intense		1.03	0.95		0.54	0.62
**Geographical area**						
North-west (ref.)			1			1
North-east			0.94			1.00
Centre			0.95			0.53*
South and islands			0.71**			1.09
**Socio-occupational class**						
Bourgeoisie or middle class (ref.)			1			1
Manual workers			1.29**			1.67*
**First-stage F-statistic**	116.82	112.81	70.44	17.90	14.19	12.50
**J-test (p)**	0.142	0.148	0.152	0.321	0.464	0.675

*Notes: IV-Poisson GMM model (Eq. 1), including*${\text{DistR}}_{i}$*as endogenous variable and two instruments:*${\text{DistE}}_{i}$ and ${\text{DistN}}_{i}\text{,}$*difference between actual age and age of eligibility for early and normal retirement*.

* p < 0.05, ** p < 0.01.

Subjects who, at the baseline interview, reported a prior diagnosis of myocardial infarction, angina pectoris, other cardiac diseases, or stroke were excluded from the analysis as prevalent cases. Prevalent cases were identified based on self-reported information available in the survey, as the linkage with administrative health records started only in 2005.

The dataset is structured as a yearly panel, with individuals being monitored up to the occurrence of their first CVD event or until the conclusion of the health follow-up in 2014.

### 2.3 Measures

The health outcome is the rate of incident CVD events. It was derived from administrative health data on hospitalisation and mortality. Follow-up is from 2005 (year of interview) to the end of 2014. Due to the annual panel structure of our dataset, the health outcome variable only takes the value one in the year of the first hospital admission (by ICD-IX codes 390–453) or death due to CVD (by ICD-X codes I00–I82), otherwise it was set to zero. The person-years used as the denominator in the rate are calculated from 2005 to the occurrence of the event or, for individuals without CVD, to the end of follow-up.

Our treatment variable is “years spent in retirement” which is defined as the time-varying number of years elapsed since labour market withdrawal. The variable is constructed as in, for example, Mazzonna and Peracchi (2012), as the positive difference between actual age (time-varying) and the age at retirement (constant): ${\text{DistR}}_{it}=$; $\max\;(0,{\text{Age}}_{it}-{R\_age}_{i}$).

We exploit the richness of the dataset to construct a wide array of **control variables** to adjust for baseline differences in terms of socioeconomic position, behavioural risk factors for CVD, and health status, following operationalisations adopted by previous studies that used ILS data (Petrelli et al., 2022; Strippoli et al., 2021). Age, included in years, was treated as a time-varying variable (i.e., increasing by one year for each year passed into the follow-up). To control for differences in risk behaviours, we include level of leisure-time physical activity (none, light, regular or intense), Body Mass Index (BMI) (normal or underweight, overweight, obese), smoking behaviour (never smokers *vs* categories of pack-years smoked during life among current or former smokers). Health status is controlled for by means of the Physical Component Summary (PCS) score, calculated based on the answers given to the SF-12 questionnaire (Apolone et al., 2005), where an increase in the value corresponds to better physical health. Among the socio-economic control variables, the following were included: geographical macro area of residence (North-west, North-east, Centre, South, and Islands) and socio-occupational class, defined based on the simplified version of the Schizzerotto's categorization (Schizzerotto, 1993) (two classes: bourgeoisie plus middle class; manual workers).^2^

Finally, a Job-Exposure Matrix (JEM) constructed from the Italian O*NET database was utilised to assign ergonomic conditions to workers in the study.^3^ This JEM assigns a specific score of exposure to many ergonomic factors at work to each 3-digit occupational code which is classified according to the Italian Statistical Office Classification of Professions (CP-2011). An Ergonomic Index was constructed that averaged scores of 17 factors potentially involving high physical workload (Fontana et al., 2022), including items focusing on force exertion, activity level, and repetitive movements of the upper limb, unfavourable postures, activities involving the whole body, and exposure to vibration. For all 17 factors, good concordance has been shown against the same items of a corresponding O*NET JEM created in the United States (d'Errico et al., 2022).

## 3 Methods

### 3.1 Endogeneity of retirement

The endogeneity of retirement presents a significant empirical challenge when attempting to determine the impact of retirement on health. Retirement is a personal decision influenced by various individual factors such as characteristics, preferences, and beliefs. One particularly problematic factor is the individuals' health status prior to retirement, as this can greatly influence their health after retirement. Additionally, there are other unobserved factors that can introduce omitted variable biases. These factors may include the nature of the job, including its physical and psychosocial workload, as well as contextual and household factors such as financial considerations, the retirement status of the individual's partner, the presence of dependent children in the household, and caregiving obligations. Previous studies have shown that these factors are associated with early retirement (De Preter et al., 2013; Hasselhorn and Apt, 2015).

Assessing the effect of retirement on health in a regression setup, without considering the correlation between the retirement choice and these factors, may lead to biased estimates of the retirement effect, due to reverse causality or omitted variable bias. Therefore, as is common practice in the field of ageing studies that investigate the consequences of retirement, we rely on an instrumental variable strategy that exploits quasi-experimental variation in the retirement choice induced by factors independent from individual health, i.e., the statutory qualifying conditions for claiming statutory early or old age pension (e.g. Angelini et al., 2009; Mazzonna and Peracchi, 2017).

### 3.2 IV-Poisson GMM model

The outcome variable of our regression equation is dichotomous and turns on during the follow-up period from 2005 to 2014, when persons have their first CVD event. The endogenous variable of interest (${\text{DistR}}_{it}\text{,}$ years spent in retirement) is continuous, greater than or equal to zero and increases with time until the end of follow up or the occurrence of a CVD event. This structuring has steered the methodology towards the use of an IV-Poisson model with continuous endogenous covariates (Mullahy, 1997; Windmeijer and Santos Silva, 1997; Wooldridge, 2010). This model, also known as ‘IV-Poisson GMM’, allows for endogeneity to be dealt with in count data models by using an instrumental variable approach, based on generalised method of moments, and two-stage estimation methods for Poisson regression models in a specification where unobserved heterogeneity is correlated with the regressors.^4^

In our estimation, we closely follow the approach of Angelini et al. (2009) and Mazzonna and Peracchi (2012, 2017), and we instrument years spent in retirement with years in eligibility (as also proposed by Battistin and Rettore, 2008). Our instrument is defined as the positive difference between actual age of individual i at time t, ${\text{Age}}_{it}\text{,}$ and the first legislated age of eligibility for early and normal retirement, $E_{i}$ and $N_{i}$ respectively, i.e., ${\text{DistE}}_{i}=$ $\max\;(0,{\text{Age}}_{it}-E_{i}$) and ${\text{DistN}}_{i}=\max\;(0,{\text{Age}}_{it}-N_{i}$). This variation can be used to predict years in retirement while being orthogonal to individual pre-retirement characteristics, as the statutory eligibility age varies across individuals depending on their birth cohort and gender due to changes in the Italian pension system over the years. For this study, we used the eligibility ages described by Angelini et al. (2009, page 504) and reported in Appendix Table 1 and2.^5^

We estimate the following equation:

Eq 1 $$y_{it}=\exp\left(\beta_{0}+\beta_{1}{DistR}_{it}+{\beta_{n}W}_{it}^{n}+o_{J}^{\beta }\right){+\epsilon }_{it}$$

Where ${\text{DistR}}_{it}$ is the endogenous variable (years elapsed since retirement), $W_{it}^{n}$ represents the vector of *n* control variables which, in the fully adjusted specification, includes age, PCS, BMI, physical activity, smoking, occupational social class prior to retirement, and geographical area of residence; $o_{J}^{\beta }$ is the offset variable (logarithm of person years); and $\epsilon_{it}$ represents the additive unit-mean errors.

Given the error function $u=y_{it}-\;\exp\left(\beta_{0}+\beta_{1}{DistR}_{it}+{\beta_{n}W}_{it}^{n}+o_{J}^{\beta }\right)$, i.e., the sample counterpart of ϵ_it_, and the set of instrumental variables ${\text{DistN}}_{i}$ and ${\text{DistE}}_{i}$ (years elapsed since eligibility for normal and early retirement), the population-moment conditions for GMM estimation are:

Eq 2 $$E\left\{{\tilde{z}}_{i}u\left(y_{it},{DistR}_{it},W_{it},\beta_{1},\beta_{2}\right)\right\}=0$$

Where the vector ${\tilde{z}}_{i}$ is partitioned as (${\text{DistN}}_{i},{\text{DistE}}_{i},W_{it}^{n}$).

Our model is over-identified because we have one endogenous regressor (years in retirement, ${DistR}_{it}$) and two instruments (years in eligibility for early and normal retirement, ${\text{DistN}}_{i}\;\text{and}\;{\text{DistE}}_{i}\text{,}$ respectively). This allows us to test, although imperfectly, the exogeneity of the instruments. Typically, this assumption in the context of IV-GMM, is assessed using a Sargan-Hansen J-test of over-identifying restrictions (Arellano, 2002). The test provides supporting evidence but does not guarantee the validity of the IV exclusion restriction assumption. Considering the poor test properties, some authors have advocated for a conservative approach in interpreting the p-value of the overidentification J-test, recommending choosing thresholds above the conventional significance levels of 5% or 10% (Kiviet and Kripfganz, 2021).

The relevance of the instruments is the other fundamental issue to consider, as instruments that are only weakly correlated with the endogenous variable may affect the finite sample properties of the IV estimates and bias them towards OLS (Bound et al., 1995). To the best of our knowledge, no test statistics has yet been proposed specifically designed to assess the relevance of the instruments in the context of the IV-Poisson GMM. Hence, we follow the previous literature using the IV-Poisson GMM methodology (see for example: Balsmeier et al., 2014; Kjøllesdal et al., 2018; Hwang, 2020), and we also assess the relevance of the instruments using the standard Kleibergen-Paap F-statistic (Kleibergen and Paap, 2006). In practise, we calculated the Kleibergen-Paap F-statistic by estimating a first stage linear regression of the endogenous variable (years in retirement) on the two instruments (${\text{DistN}}_{i}$ and ${\text{DistE}}_{i}$) in the base year (i.e. 2005), including the full set of controls.

In our primary analysis, we evaluated three distinct models, each with a successive level of adjustment. Model 1 adjusted for age alone. Model 2 expanded on this by including additional controls for physical activity, BMI, smoking habit, and the physical health indicator PCS derived from the SF-12 questionnaire. Model 3 further incorporated socioeconomic variables, such as geographical area of residence and a variable distinguishing manual workers. Factors like physical activity, smoking, and BMI, which research has shown can be influenced by retirement (e.g., Eibich, 2015; Insler, 2014), may be part of the causal pathway between retirement and CVD. By incrementally adding these variables to the simpler Model 1, we aim to discern their potential as either confounders or mediators.

Estimated coefficients are exponentiated and reported as Incidence Rate Ratios (IRR) (Hwang, 2020). All analyses are run separately for men and women.

### 3.3 Heterogeneity and sensitivity analyses

We performed additional analyses to shed light on the potential mechanisms that could explain our results, by running a set of sensitivity analyses in which occupational social class (bourgeoisie + middle class *vs* manual workers) and ergonomic index (divided into two groups, low and high physical workload, according to the median value) were tested as effect modifiers of the association between years in retirement and CVD incidence. First, we ran a set of separate stratified regressions on each of these four subpopulations. Running separate regressions has the advantage that we can model the association between each covariate and the outcome, allowing for greater flexibility in terms of the influence that they can have on CVD outcomes, without imposing the same functional form among different occupational groups and workers exposed to different levels of physical work. Second, for the analyses in which we find suggestion of heterogeneous effects of retirement years on CVD, we also estimate the following specification, by expanding equation 2 with the introduction of an interaction term:

Eq 3 $$y_{it}=\exp\left(\beta_{0}+\beta_{1}{DistR}_{it}+\gamma {DistR}_{it}\times X_{i}+{{\tilde{\beta }}_{n}\tilde{W}}_{it}^{n}+o_{J}^{\beta }\right){+\epsilon }_{it}$$

Where ${DistR}_{it}\times X_{i}$ is an interaction term between ${DistR}_{it}$ and effect modifiers (i.e., occupational social class and ergonomic index are all treated as dichotomous variables). ${\tilde{W}}_{it}^{n}$ is the vector of control variables introduced above (fully adjusted specification). Following Wooldridge (2010), both years elapsed since eligibility for normal and early retirement, ${\text{DistN}}_{i}$ and ${\text{DistE}}_{i}$, and their interaction terms, ${\text{DistN}}_{i}\times X_{i},{\text{DistE}}_{i}\times X_{i}$, were included as instrumental variables. ${DistR}_{it}$ effect modification on CVD incidence was ascertained by checking the statistical significance of the γ coefficient.

To confirm the robustness of our results, we conducted a series of checks. These examined how sensitive our findings were to variations in key variables, such as the number of years spent in retirement and the severity of cardiovascular disease outcomes. We also scrutinized the influence of crucial potential confounders, including age, cohort effects, and baseline health status.

## 4 Results

### 4.1 Main results

The sample, which is described in Table 1, consists of 4061 men and 1958 women, whose baseline mean age was 68.2 and 70.4 years, respectively. During the follow-up, 1200 CVD events were observed among men and 457 among women.

Table 2 shows CVD's estimated IRR which is associated with the number of years spent in retirement ($DistR_{it}$) from various IV Poisson GMM regressions, using $DistE_{it}$ and DistN as instruments for $DistR_{it}$. The table also shows the first stage Kleibergen-Paap F-statistic for the joint significance of the excluded instruments and the Sargan-Hansen J-test p-value. First-stage regression results are available in Supplementary Data Table 3.

**Table 3:** Incidence rate ratios (IRR) and 95% Confidence Interval of cardiovascular diseases (CVD) associated with years in retirement: IV Poisson GMM, by socio-occupational class (Men).

	Men	
	High class	Low class
	IRR	IRR
$DistR_{it}$ Years in retirement	0.93	0.79**
(95% CI)	(0.74–1.18)	(0.68–0.90)
Number of subjects	1013	3048
Number of events	264	936
**First-stage F statistic**	24.60	37.57
**J-test (p)**	0.545	0.134

*Notes: IV-Poisson GMM model with years in retirement as endogenous variable and two instruments:*${\text{DistE}}_{i}$ and ${\text{DistN}}_{i}$*(Eq. 1, fully adjusted). High class = bourgeoisie and middle class. Low class = working class doing manual work.*

*p < 0.05, ** *p* < 0.01.

Our analyses confirm that statutory eligibility ages are a relevant determinant of retirement choices and that they are plausibly exogenous instruments for the time spent in retirement. The first-stage F-statistic is above 70 in all models for men, indicating that the distance from the eligibility age is a strong predictor of the time spent in retirement. For women, the estimated F-statistic varies between 12 and 17, indicating that instruments are still relevant but weaker than for men. It is possible that only a minority of women were affected by the eligibility rules due to concurrent family and caring responsibilities, which may have led to a relatively higher proportion of them retiring regardless of having reached pension eligibility. The p-values of the Sargan-Hansen J-test ranged between 0.14 and 0.16 for men and from 0.32 to 0.67 for women, thereby suggesting that $DistE_{it}$ and ${\text{DistN}}_{\text{it}}$ are plausibly exogenous instruments. Further supporting evidence for the validity of the instruments is provided by the sensitivity and stratified analyses (Supplementary Tables 10–11), where the p-values of the J-test become even higher (above 0.50 for men).

Results from Model 1 (adjusted only for age) show a significant decrease in CVD incidence associated with years spent in retirement in both genders, with a stronger association in women than men (IRR = 0.80 and IRR = 0.65 for each additional year among men and women, respectively). Incorporating lifestyle and health indicators (Model 2) or adding occupational social class and geographical variables (Model 3) only slightly diminished the observed associations. However, the protective effect of a longer retirement on CVD risk remained substantial and statistically significant for both genders. The minimal changes in the incidence rate ratio after adjusting for BMI or healthy lifestyle factors indicate that they do not serve as significant confounders or mediators in the relationship between retirement duration and CVD within our study context.

### 4.2 Results from the heterogeneity analysis

In stratified analyses among men, we observed more robust associations between CVD incidence and years in retirement for those in lower occupational classes (as shown in Table 3) and those with higher exposure to physical workload (as detailed in Table 4). For these groups, each additional year of retirement is associated with a significant 20% decrease in CVD incidence. Conversely, for men in higher occupational classes or those with jobs entailing low physical workload, the association, while still protective, was not statistically significant. The limited number of CVD events in the high occupational class group may have affected the statistical power, yet this issue did not arise in the low physical workload group, which was comparable in size to the high workload group. Nonetheless, when we applied Equation (3), no significant interaction was found between years in retirement and occupational social class (p-value = 0.47) or exposure to ergonomic factors (p-value = 0.38) (Supplementary Data Tables 4–5). Hence, we have compelling evidence that the beneficial effects of time spent in retirement on CVD are more pronounced among men who were previously employed in lower occupational classes and in jobs with high physical workload. For those in the higher occupational categories, the evidence is less clear-cut and less precise. Even so, the interaction model hints at a possibly weaker, yet still protective, effect of retirement on CVD for these more advantaged workers as well.

**Table 4:** Incidence rate ratios (IRR) and 95% Confidence Interval of cardiovascular diseases (CVD) associated with years in retirement: IV Poisson GMM, by ergonomic index (Men).

	Men	
	Low Physical Workload	High Physical Workload
	IRR	IRR
$DistR_{it}$ Years in retirement	0.90	0.81*
(95% CI)	(0.71–1.14)	(0.67–0.98)
Number of subjects	1605	1605
Number of events	465	492
**First-stage F statistic**	35.83	19.45
**J-test (p)**	0.707	0.088

*Notes: IV-Poisson GMM model with years in retirement as endogenous variable and two instruments:*${\text{DistE}}_{i}$ and ${\text{DistN}}_{i}$*(Eq. 1, fully adjusted). Low/High Physical Workload = Ergonomic index below/above the median. Missing: 851 subjects*.

* *p* < 0.05, ** *p* < 0.01.

Based on the analysis conducted, the findings for women did not provide conclusive evidence of heterogeneities along the dimensions mentioned. Like the results for men, the stratified analyses showed a significant protective effect of years of retirement only for women employed in manual occupations and occupations with high physical workload. However, it is important to note that the relevance and exogeneity tests of the instruments did not support the validity of the instrumental variable (10.13039/501100000026IV) estimation in these subgroups, so the results should be interpreted with caution. Additionally, the model did not converge for the low physical workload category, and the interaction models did not pass the instrumental variable diagnostics. Please refer to the Supplementary Tables 6–9 for more detailed information about the results.

### 4.3 Results from the sensitivity analysis

The sensitivity analyses support the results for men quite strongly and consistently (Supplementary Table 10, models 1–9), whereas, among women, as for the subgroup analysis, the results seem to be very sensitive to changes to the main specification, and the protective effect of time in retirement persisted only in few tests (Supplementary Table 11, models 3, 8, 9).

Focusing on men, the following patterns emerge. First, using a more flexible age specification by controlling for polynomials of age (a quadratic term in Model 2 and a cubic term in Model 3), the IRRs for CVD remain significant and do not change substantially in magnitude. This consistency indicates that our initial linear age specification was reasonably effective in adjusting for the increasing risk of CVD with advancing age. These findings are further supported by analyses using the logarithm of age and age-fixed effects.

A potential concern is that our IV strategy and model specification may not adequately account for pre-existing health disparities. However, when we expand our analysis to include chronic morbidity in Model 4—utilizing the Chronic Morbidity Index, which is a continuous index based on self-reported information about 22 health conditions from the baseline survey—we find that the IRR for time spent in retirement remains consistent with the main model (an IRR of 0.82 compared to 0.83). This is despite the fact that the CMI itself is significantly associated with the outcome (an IRR of 1.01, p < 0.01).

Moreover, we assess whether differences across cohorts may be driving the results. Adding explicitly cohorts to the model is problematic, given that we are already including in the model age, and that the principal source of variability of our instrumental variables is between cohorts. However, despite the high correlation between these variables, the inclusion of linear cohort among the controls (as well its polynomial transformations or fixed effects) leaves the point estimate quite unchanged (Model M5, IRR 0.86), at the expenses of substantial widening of the confidence interval.

As it seems plausible that relief from strenuous working conditions might have different short- and long-term effects, we explore more flexible specifications for the time spent in retirement and examine the effects of different lengths of retirement. In models M6 and M7, using 5 years as the cut-off (25th percentile of DistR), we found that the reduction in CVD risk is greater when the duration of retirement is greater than 5 years, a trend that was confirmed for different thresholds up to 9 years.

Finally, we provide evidence that the outcome definition does not drive the results by retaining also prevalent CVD cases in the sample and by restricting the CVD risk to its most severe manifestation, CVD mortality and CVD hospitalisation of long duration, defined by a number of hospitalisation days in the upper tertile of the distribution. The results confirm the validity of the IV strategy and show that, even in these specifications, time spent in retirement significantly reduces the risk of CVD (models M8-M9).

## 5 Discussion

This study, conducted in a large Italian cohort of retirees, shows that years spent in retirement reduce the incidence of CVD in both men and women. CVD was assessed using certified administrative information on hospitalisations and mortality. The results, at least for men, can be interpreted in a causal manner because they were obtained using an instrumental variable strategy embedded in a Poisson model, and the evidence from both the various IV diagnostics and sensitivity checks confirms their validity and robustness.

In fully adjusted models, the risk of CVD decreased by 17% for each year of retirement for men, while the decrease was almost double (29%) for women, providing evidence of a beneficial and protective effect of longer retirement on CVD in both sexes. When we further disaggregate our sample by occupational social class or ergonomic conditions, the analyses for men reveal a social gradient, with blue-collar workers and workers exposed to high physical demands showing a greater benefit from each year of retirement. For women, although the results for the full sample are in line with those for men, the limited sample size may have reduced the ability to draw conclusions about possible heterogeneities. Conversely, it may be that the heterogeneous effect of retirement on health runs through individual and contextual dimensions other than work-related exposures for women, that are not well captured by variables commonly available in empirical studies, such as the need to combine paid and unpaid work or to provide care for sick or disabled relatives (Carrino et al., 2023). Moreover, the fact that the results for women were not confirmed in the various sensitivity analyses means that conclusions can only be tentative, suggesting that time spent in retirement may also be beneficial for women, but that the results are unstable and should be treated with caution.

The association between retirement and CVD appears to be mixed in the literature, as concluded in a recent systematic review in which results from longitudinal studies were found to be quite heterogeneous and geographically clustered (Xue et al., 2020). However, given the endogeneity of the retirement decision with respect to health status, the estimates of most studies that use traditional regression techniques included in this review may have been affected by residual confounding with respect to health. There are a few studies on retirement and CVD risk with a causal design to which we can refer. Our findings are consistent with Ardito et al. (2020), who used an IV strategy to show that delaying retirement in Italy increased the risk of CVD hospitalisation for men. Nonetheless, other IV studies focusing on CVD found no significant association between retirement and different CVD-related outcomes, such as myocardial infarction (Coe and Lindeboom, 2008), CVD mortality (Bound and Waidmann, 2007), or CVD hospitalisation (Grøtting and Lillebø, 2020; Serrano‐Alarcón et al., 2023). Only one study using data from the English Longitudinal Study of Aging found a significant increase in CVD incidence associated with retirement (Behncke, 2012) when a matching estimator was used, but the effect became null when the IV strategy was used again. However, in this study, the CVD outcome was self-reported, with the possibility that retired individuals may have over-reported such conditions. A possible explanation for the null effect of retirement on CVD in these studies could be that their follow-up was too short. Indeed, while the study by Ardito et al. (2020), had a follow-up for CVD detection of about 10 years, similar to this paper, the other studies mentioned above focused on short-term effects of retirement (Bound and Waidmann, 2007; Behncke, 2012; Grøtting and Lillebø, 2020; Serrano‐Alarcón et al., 2023) or medium-term effects, with data on CVD only up to 4 years after retirement (Coe and Lindeboom, 2008).

The results of this study support the idea that workers with a low occupational social class and a high physical workload may benefit more from time in retirement. This is consistent with previous research showing that retirement has a positive effect on self-rated physical and mental health and a negative effect on frailty, particularly for manual workers or those in physically or psychosocially demanding jobs (Eibich, 2015; Carrino et al., 2020; Westerlund et al., 2009; Matthews, 2014; Kalousova and de Leon, 2015). In this vein, our results speak to those of Ardito et al. (2020), which showed that delayed retirement poses no risk for white collars’ CVD, whereas it increases the risk of CVD for manual workers. It is also coherent with Serrano‐Alarcón et al. (2023), who found that an Italian reform that raised the retirement age increased the risk of hospitalisation for injuries and mental disorders, particularly among more vulnerable workers. Contrary to this range of studies, the sole existing review on the topic by Schaap et al. (2018), which considered only eight studies, found that socioeconomic position does not significantly modify the relationship between retirement and health. Given this, there is a clear need for additional research to more comprehensively understand how retirement and related policies might have differentiated health impacts across socio-economic groups.

Finally, given the large size of our estimates, one might reasonably expect the health effects of the reforms to be detectable in the aggregate data. However, this is unlikely for two main reasons: the affected population has a limited weight in the total population, and individuals may respond to a tightening of pension eligibility by taking alternative paths to retirement, at least partly diluting the full potential treatment effect of the policy (Gruber and Wise, 2007). Nevertheless, national age-adjusted CVD incidence statistics show that the declining risk trend has levelled off since 2011 (GBD, 2019). Although these changes cannot be interpreted causally, they are consistent with the increase in the retirement age implemented in pension reforms since the 1990s.

## 6 Conclusions

Governments around the world, responding to the pressures of ageing and declining fertility rates, have begun to reform pension systems by tightening eligibility conditions, with the aim of encouraging higher employment among older workers and reducing pension expenditure. This has raised concerns about the sustainability of extending the working lives of ageing workforces, which are on average characterised by poorer health, reduced working capacity and employability.

In the present study, we have attempted to contribute to this debate by assessing the impact of time spent in retirement on the risk of CVD, indirectly testing whether reforms aimed at delaying retirement might have unintended health effects on older workers. In our analysis, based on an instrumental variables’ strategy embedded in a Poisson model, we observed a significant reduction in CVD associated with more years in retirement for both sexes. The protective effect of retirement is driven by male retirees who, prior to retirement, were employed in jobs characterised by more physically demanding working conditions and lower skill endowments.

It is natural to speculate whether our estimates have implications for the advisability of raising the early or normal retirement age. Our results suggest that national policymakers planning to tighten eligibility requirements need to consider that such policies may lead to an increased burden of CVD in the older population. The protective effect of years in retirement on the development of CVD found among workers with poorer ergonomic conditions suggests that an increase in the retirement age does not mean the same thing for all categories of workers.

In Italy, as in around two-thirds of European countries, legislation already exists to enable earlier retirement for those working in strenuous and hazardous jobs (Natali et al., 2016). However, the current evidence suggests the need for a more thorough policy discussion. This should concentrate on potentially broadening the range of occupations qualifying for early retirement and introducing more flexible pension arrangements. It is crucial that policy efforts include a range of support measures aimed at making it sustainable for people to work longer and to adapt to higher mandatory retirement ages. Greater consideration could be given to inclusive labour market policies for older workers that facilitate workplace adaptations, a gradual transition to retirement, and differentiated pension eligibility conditions and benefits that could help compensate for existing socioeconomic differences in health and longevity (OECD, 2017). However, even flexible compensation is ethically questionable as it presupposes the maintenance of health differentials, which are in fact inequalities. Pension reforms can no longer be based on an unconditional increase in the retirement age. Reforms should increasingly be designed in an integrated way with other social reforms.

## Ethics approval and consent to participate

The study was performed using anonymized data of the Italian Longitudinal Study, which has been developed and is maintained within the framework of the National Statistics Plan (NSP Code: IST-02566), through an agreement between ISTAT, Italian Ministry of Health and the Piedmont Region, and it has been approved by the Italian Data Protection Authority for privacy. The study was conducted according to the principles expressed in the Declaration of Helsinki for studies on humans. Written informed consent has been obtained from all participants.

## Funding

This study was funded by the research grant n. RF-2019-12370893, from the 10.13039/501100003196Italian Ministry of Health (granted to Giuseppe Costa and Angelo d’Errico).

## CRediT authorship contribution statement

**Dario Fontana:** Writing – review & editing, Writing – original draft, Formal analysis, Data curation, Conceptualization. **Chiara Ardito:** Writing – review & editing, Writing – original draft, Validation, Supervision, Methodology, Funding acquisition, Conceptualization. **Roberto Leombruni:** Writing – review & editing, Supervision, Conceptualization. **Elena Strippoli:** Writing – review & editing, Methodology. **Angelo d’Errico:** Writing – review & editing, Writing – original draft, Supervision, Project administration, Methodology, Funding acquisition, Conceptualization.

## Declaration of competing interest

The authors declare that they have no competing interests.

## Data Availability

The authors do not have permission to share data.

## Footnotes

1 For more details on ILS data, see Marinacci et al. (2013) and Strippoli et al. (2021).

2 Although information on educational attainment was available, it was not included in the regressions together with occupational social class because the two variables were highly correlated (Spearman's rho 0.60 among men and 0.50 among women).

3 We used the ergonomic index variable only as a stratification variable and not as a control, as it is highly correlated with occupational social class (Spearman's rho = 0.69 among men and = 0.67 among women).

4 We implemented the estimation with the STATA 17 command “ivpoisson” (https://www.stata.com/manuals/rivpoisson.pdf).

5 Eligibility for statutory pension also depends on years of contributions, but this information is not available in our data. Therefore, we only use changes in the minimum age for claiming a pension.
