# Supplementary material for: Does the time spent in retirement improve health? An IV-Poisson assessment on the incidence of cardiovascular diseases
Source: Soc Sci Med. 2024 Aug;354:117084. doi: 10.1016/j.socscimed.2024.117084 (PMC11364157; doi:10.1016/j.socscimed.2024.117084)
Supplement: Multimedia component 1 [file mmc1.pdf]

Supplementary data to the manuscript

"Does the time spent in retirement improve health? An IV-Poisson assessment on the incidence of cardiovascular diseases"

Supplementary table 1 – Minimum age for old age pension (Angelini et al., 2009).

Period	Private sector		Public sector	
	Men	Women	Men	Women
from 1961 to 1993	60	55	65	60
1994	61	56	65	60
1995	61.5	56.5	65	60
1996	62	57	65	60
1997	63	58	65	60
1998	63.5	58.5	65	60
1999	64	59	65	60
from 2000 to 2007	65	60	65	60

Supplementary table 2 – Minimum age for early retirement pension (Angelini et al., 2009).

Period	Private sector		Public sector	
	Men	Women	Men	Women
from 1961 to 1964	no early retirement			
from 1965 to 1995	35 years of contributions		25 years of contributions	
from 1996 to 1997	52	52	52	52
1998	54	54	53	53
1999	55	55	53	53
2000	55	55	54	54
2001	56	56	55	55
2002	57	57	55	55
2003	57	57	56	56
from 2004 to 2007	57	57	57	57

Supplementary table 3 – First-Stage Results: Ordinary Least Squares Estimates of the Effect of the Instruments ($DistN_{it}$, $DistE_{it}$) and additional controls on 'Years spent in retirement', by gender

	**Men** (N 4,061)			**Women** (N 1,958)		
	**Model 1**	**Model 2**	**Model 3**	**Model 1**	**Model 2**	**Model 3**
	**Coefficient**	**Coefficient**	**Coefficient**	**Coefficient**	**Coefficient**	**Coefficient**
$DistN_{it}$ Normal retirement	0.19**	0.19**	0.18**	0.21**	0.22**	0.22**
(95% CI)	(0.12-0.25)	(0.12-0.26)	(0.11-0.24)	(0.07–0.35)	(0.07–0.37)	(0.08–0.37)
$DistE_{it}$ Early retirement	0.42**	0.45**	0.41**	0.22**	0.22**	0.24**
(95% CI)	(0.36-0.49)	(0.39-0.52)	(0.34-0.48)	(0.15–0.29)	(0.14–0.30)	(0.14–0.33)
**Age**	0.07	0.05	0.10	0.45**	0.44**	0.42**
**BMI**						
Normal weight or underweight (ref.)		1	1		1	1
Overweight		-0.12	-0.09		0.14	0.17
Obese		0.38	0.39		0.25	0.26
**PCS**		-0.01	-0.01		-0.01	-0.02
**Pack-years of smoking**						
0 never smokers (ref.)		1	1		1	1
0.1 - 20		0.58**	0.56**		0.17	0.11
> 20		-0.39	0.08		0.18	0.20
**Leisure-time physical activity**						
No activity (ref.)		1	1		1	1
Light		0.83**	0.58**		0.36	0.28
Regular or intense		1.35**	0.77**		0.52	0.20
**Geographical area**						
North-west (ref.)			1			1
North-east			-0.62**			-0.21
Centre			-1.62**			-1.16**
South and islands			-2.77**			-1.14**
**Socio-occupational class**						
Bourgeoisie or middle class (ref.)			1			1
Manual workers			0.86**			0.60*
**First-stage F statistic**	116.82	112.81	70.44	17.90	14.19	12.50

** p<0.05, ** p<0.01.*

Supplementary table 4 – Interaction model by socio-occupational class (Men)

	IRR	p
$\boldsymbol{DistR_{it}}$ Years in retirement	0.84	0.002
(95% CI)	(0.76-0.93)	
**Socio-occupational class**		
Bourgeoisie or middle class (ref.)	1	
Manual workers	0.95	0.885
(95% CI)	(0.58-1.57)	
**Interaction term**	0.98	0.467
(95% CI)	(0.94-1.03)	
**J-test**		0.100

Note: Incidence Rate Ratios (IRR) of cardiovascular diseases (CVD) from a IV-Poisson GMM model with years in retirement as endogenous variable (instrumental variables: difference between actual age and age of eligibility for early and normal retirement), adjusted for age, physical activity, BMI, smoking habits, Physical Component Summary (PCS), geographical area and socio-occupational class, with an interaction term between socio-occupational class and years in retirement (Eq. 3, fully adjusted model).

Supplementary table 5 – Interaction model by ergonomic index (median as cut-off) (Men)

	IRR	p
$\boldsymbol{DistR_{it}}$ Years in retirement	0.86	0.048
(95% CI)	(0.75-0.99)	
**Ergonomic Index**		
Low Physical Workload	1	
High Physical Workload	1.26	0.333
(95% CI)	(0.77-2.02)	
**Interaction term**	0.98	0.388
(95% CI)	(0.95-1.22)	
**J-test**		0.171

Note: Incidence Rate Ratios (IRR) of cardiovascular diseases (CVD) from a IV-Poisson GMM model with years in retirement as endogenous variable (instrumental variables: difference between actual age and age of eligibility for early and normal retirement), adjusted for age, physical activity, BMI, smoking habits, Physical Component Summary (PCS), geographical area and physical workload, with an interaction term between physical workload and years in retirement (Eq. 3, fully adjusted model. Missing records: 851 subjects).

Supplementary Table 6 – Incidence rate ratios and 95% Confidence Interval of cardiovascular diseases (CVD) associated with years in retirement: IV Poisson GMM, by socio-occupational class (Women).

	Women	
	**High class**	**Low class**
	**IRR**	**IRR**
$\boldsymbol{DistR_{it}}$ Years in retirement	1.11	0.62**
(95% CI)	(0.52-2.39)	(0.58-0.68)
Number of subjects	356	1,062
Number of events	64	393
**First-stage F statistic**	2.77	9.95
**J-test (p)**	0.541	<0.001

Notes: IV-Poisson GMM model with years in retirement as endogenous variable (instrumental variables: difference between actual age and age of eligibility for early and normal retirement) and adjusted for: age, physical activity, BMI, smoking habits, Physical Component Summary (PCS), geographical area and manual work (Eq. 1, fully adjusted model). High class = bourgeoisie and middle class. Low class = working class doing manual work.

** p<0.05, ** p<0.01.*

Supplementary Table 7 – Incidence rate ratios and 95% Confidence Interval of cardiovascular diseases (CVD) associated with years in retirement: IV Poisson GMM, among women, by ergonomic index (Women).

	Women	
	**Low Physical Workload**	**High Physical Workload**
	**IRR**	**IRR**
$\boldsymbol{DistR_{it}}$ Years in retirement	Hessian is not positive semidefinite	0.59**
(95% CI)		(0.50-0.69)
Number of subjects	831	831
Number of events	181	207
**First-stage F statistic**	-	5.85
**J-test (p)**	-	0.028

Notes: IV-Poisson GMM model with years in retirement as endogenous variable (instrumental variables: difference between actual age and age of eligibility for early and normal retirement) and adjusted for: age, physical activity, BMI, smoking habits, Physical Component Summary (PCS), geographical area and manual work (Eq. 1, fully adjusted model. Missing records: 296 subjects). Low / High Physical Workload = Ergonomic index below / above the median.

** p<0.05, ** p<0.01.*

Supplementary table 8 – Interaction model by socio-occupational class (Women).

	IRR	p
$\boldsymbol{DistR_{it}}$ Years in retirement	0.71	0.000
(95% CI)	(0.61-0.84)	
**Socio-occupational class**		
Bourgeoisie or middle class (ref.)	1	
Manual workers	1.88	0.181
(95% CI)	(0.74-4.76)	
**Interaction variable**	0.90	0.062
(95% CI)	(0.80-1.01)	
**J-test**		0.665

Note: Incidence Rate Ratios (IRR) of cardiovascular diseases (CVD) from a IV-Poisson GMM model with years in retirement as endogenous variable (instrumental variables: difference between actual age and age of eligibility for early and normal retirement), adjusted for age, physical activity, BMI, smoking habits, Physical Component Summary (PCS), geographical area and socio-occupational class, with an interaction term between socio-occupational class and years in retirement (Eq. 3, fully adjusted model).

Supplementary table 9 – Interaction model by ergonomic index (Women).

	IRR	p
$\boldsymbol{DistR_{it}}$ Years in retirement	2.11	0.013
(95% CI)	(1.17-3.82)	
**Ergonomic Index**		
Low Physical Workload	1	
High Physical Workload	1.86	0.238
(95% CI)	(0.66-5.26)	
**Interaction variable**	0.80	0.285
(95% CI)	(0.94-1.01)	
**J-test**		0.459

Note: Incidence Rate Ratios (IRR) of cardiovascular diseases (CVD) from a IV-Poisson GMM model with years in retirement as endogenous variable (instrumental variables: difference between actual age and age of eligibility for early and normal retirement), adjusted for age, physical activity, BMI, smoking habits, Physical Component Summary (PCS), geographical area and physical workload, with an interaction term between physical workload and years in retirement (Eq. 3, fully adjusted model). Low / High Physical Workload = Ergonomic index below / above the median.

Supplementary Table 10 – Sensitivity analysis for **men**.
Incidence rate ratios (IRR) and 95% confidence intervals (CI) of CVD associated with years of retirement using different specifications (IV Poisson GMM models).

		CONTROLS				TREATMENT		OUTCOME	
	**Main results (Tab. 2, M. 3)**	**Quadratic in age**	**Cubic in age**	**Adding CMI**	**Adding year of birth**	**DistR <= 5 years**	**DistR > 5 years**	**Including prevalent CVD**	**Severe CVD only**
	**M1**	**M2**	**M3**	**M4**	**M5**	**M6**	**M7**	**M8**	**M9**
**Years in retirement**	0.83**	0.87**	0.82**	0.82**	0.86	0.94	0.92*	0.77**	0.78**
**(95% CI)**	(0.74-0.93)	(0.79-0.95)	(0.75-0.90)	(0.73-0.91)	(0.30-2.38)	(0.85-1.03)	(0.86-0.98)	(0.70-0.84)	(0.66-0.93)
**N. ind.**	4061	4061	4061	4061	4061	1076	2984	5034	4061
**N. events**	1200	1200	1200	1200	1200	270	930	1735	401
**First-stage F statistic**	70.44	78.71	27.24	67.56	70.34	79.64	19.53	72.47	70.45
**J-test (p)**	0.15	0.76	0.36	0.13	0.10	0.46	#	0.11	0.35

Notes: Notes: IV-Poisson GMM model specified as in the main model of Tab. 2, Model 3, unless differently specified, i.e.: models M2 and M3 substitute linear age with age polynomials (quadratic and cubic, respectively). Models M4 and M5 add to the controls the CMI (chronic morbidity index) and the year of birth, respectively. Models M6 and M7 are run on subsample stratified below/above the 25th percentile of the endogenous variable, years in retirement (DistR). M8 includes in the sample also individuals who declare at baseline having had histories of CVD before (excluded in the main analysis). M9 adopts an outcome variable taking value 1 only for the most severe cases of CVD, i.e. CVD death plus CVD hospitalization of long duration (upper tercile).

** p<0.05, ** p<0.01.*

Supplementary Table 11 – Sensitivity analysis for **women**.
Incidence rate ratios (IRR) and 95% confidence intervals (CI) of CVD associated with years of retirement using different specifications (IV Poisson GMM models).

		CONTROLS				TREATMENT		OUTCOME	
	**Main results (Mod. 3, Tab. 2)**	**Quadratic in age**	**Cubic in age**	**Adding CMI**	**Adding linear cohorts**	**DistR <= 7 years**	**DistR > 7 years**	**Including prevalent CVD**	**Severe CVD only**
	**M1**	**M2**	**M3**	**M4**	**M5**	**M6**	**M7**	**M8**	**M9**
**Years in retirement**	0.71**	0.80	0.78*	1.03	0.93	0.92	1.00	0.62**	0.57**
**(95% CI)**	(0.60–0.84)	(0.61-1.06)	(0.61-0.99)	(0.65-1.63)	(0.76-1.54)	(0.76-1.10)	(0.79-1.27)	(0.54-0.71)	(0.49-0.66)
**N. ind.**	1958	1958	1958	1958	1958	486	1472	2323	1958
**N. events**	457	457	457	457	457	73	384	648	131
**First-stage F statistic**	12.50	0.93	0.82	12.37	12.48	29.75	20.44	15.00	12.50
**J-test (p)**	0.68	0.76	0.52	0.74	0.66	0.28	#	0.67	0.01

Notes: Notes: IV-Poisson GMM model specified as in the main model of Tab. 2, Model 3, unless differently specified, i.e.: models M2 and M3 substitute linear age with age polynomials (quadratic and cubic, respectively). Models M4 and M5 add to the controls the CMI (chronic morbidity index) and the year of birth, respectively. Models M6 and M7 are run on subsample stratified below/above the 25th percentile of the endogenous variable, years in retirement (DistR). M8 includes in the sample also individuals who declare at baseline having had histories of CVD before (excluded in the main analysis). M9 adopts an outcome variable taking value 1 only for the most severe cases of CVD, i.e. CVD death plus CVD hospitalization of long duration (upper tercile).

** p<0.05, ** p<0.01.*
